# Assessing record linkage between health care and Vital Statistics databases using deterministic methods

**DOI:** 10.1186/1472-6963-6-48

**Published:** 2006-04-05

**Authors:** Bing Li, Hude Quan, Andrew Fong, Mingshan Lu

**Affiliations:** 1 Department of Community Health Sciences, University of Calgary, Calgary, Alberta, T2N 4N1, Canada; 2 Institute of Health Economics, Edmonton, Alberta, T5J 3N4, Canada; 3 Centre for Health and Policy Studies, University of Calgary, Calgary, Alberta, T2N 4N1, Canada; 4 Department of Economics, University of Calgary, Calgary, Alberta, T2N 1N4, Canada

## Abstract

**Background:**

We assessed the linkage and correct linkage rate using deterministic record linkage among three commonly used Canadian databases, namely, the population registry, hospital discharge data and Vital Statistics registry.

**Methods:**

Three combinations of four personal identifiers (surname, first name, sex and date of birth) were used to determine the optimal combination. The correct linkage rate was assessed using a unique personal health number available in all three databases.

**Results:**

Among the three combinations, the combination of surname, sex, and date of birth had the highest linkage rate of 88.0% and 93.1%, and the second highest correct linkage rate of 96.9% and 98.9% between the population registry and Vital Statistics registry, and between the hospital discharge data and Vital Statistics registry in 2001, respectively. Adding the first name to the combination of the three identifiers above increased correct linkage by less than 1%, but at the cost of lowering the linkage rate almost by 10%.

**Conclusion:**

Our findings suggest that the combination of surname, sex and date of birth appears to be optimal using deterministic linkage. The linkage and correct linkage rates appear to vary by age and the type of database, but not by sex.

## Background

Record linkage techniques are used widely in epidemiological studies to obtain comprehensive information and conduct more robust analyses [1-13]. For any record linkage project, two questions must be answered: 1) What is the best way to achieve a high linkage rate in a cost-effective manner? 2) What is the correct linkage rate among the records that are linked?

The evolution from manual to computerized record linkage has helped to answer the first question. There are two commonly used computerized record linkage approaches: deterministic and probabilistic [8,9]. The deterministic record linkage approach generates links on the basis of a full agreement of a unique identifier or a set of common identifiers. This method minimizes the uncertainties in the match between two databases since only a complete match on a set of personal variables is accepted at the cost of lowering the linkage rate. The probabilistic record linkage approach creates links between two databases based on a calculated statistical probability of a set of common identifiers. The probability is used to determine whether a pair of records approximately refers to the same individual. Thus, probabilistic linkage maximizes linkage theoretically but may result in uncertainty for some potential links. For the second question, correct linkage depends on the amount of personal identifying information available on the records being linked. [6] When enough information is available to use deterministic record linkage, this method should increase the correct linkage with little sacrifice in lowering the linkage rate. On the other hand, when unique personal identifiers (such as social insurance number or personal health number) are not available, the linkage and correct linkage rates depend heavily on the uniqueness of a set of proxies (such as name, sex and date of birth).

Using both deterministic and probabilistic linkage approaches, Roos, Wajda and Nicol [8] assessed the linkage rate between Manitoba Health Services Commission and Canadian Vital Statistics data to verify Manitoba deaths aged 25 or older during 1970 and 1979. The deterministic linkage approach was first used to link the records in the two databases using eight personal identifiers (i.e. sex, death year, death month, death day, birth year, birth month, initial and location). Then, the probabilistic record linkage approach was used to further match the remaining unlinked records after deterministic linkage in these two databases. The linkage rate increased from 80.7% in 1970 to 95.8% in 1979. Roos and Wajda [9] repeated the linkage assessment but used specific weights model methodology. The model was based on weights for eight personal identifiers (i.e., sex, death month, death day, birth year, birth month, location, initial and marital status). A numerical weight for each variable was calculated by logarithm of agreement or disagreement frequency in linked pairs divided by that in unlinked pairs. They found that with the improvement in data quality, the record linkage rate improved significantly. The deterministic linkage approach matched 98.6% of Vital Statistics records with 1987 Manitoba Health Services Commission data in contrast to 93.7% for 1973 data. Neither study took surname as an identifying variable because researchers did not have access to individual or family names.

In our study, we selected four common identifiers (surname, first name, sex, and date of birth) from a set of personal identifiers and composed three different combinations of four identifiers: (1) a combination of surname, sex and date of birth, (2) first name, sex, and date of birth, and (3) surname, first name, sex and date of birth. Then we assessed the deterministic linkage between the Vital Statistics registry and the population registry in one scenario, and between the Vital Statistics registry and the in-hospital death records in hospital discharge data in the second scenario for the fiscal years 1998/99 through 2001/02 for each combination. We assessed these three databases because they are widely used in population and health services research to determine death status, cause of death and medical history.

## Methods

### Databases

Three administrative health databases were employed. We restricted our study population to residents of Calgary Health Region (CHR), Alberta, Canada during fiscal years 1998/99 through 2001/02. As of March 2002, CHR's population was approximately 1.1 million. Infants less than one year were excluded because of missing or inaccurate variables such as name. In Vital Statistics individuals were defined as CHR residents based on Standard Geographical Classification (SGC) for assessment of the linkage between Vital Statistics and the population registry. The SGC was used to classify residential areas based address or community [14]. Of 21679 deaths occurred in the CHR during 1998/99 to 2001/2002, 99 were excluded due to unknown SGC, 1358 were also excluded because of non-CHR residents, and 20222 were finally analyzed. Residential postal codes recorded in the hospital discharge data were used to define CHR residents for assessment of the linkage between Vital Statistics and hospital discharge data.

The first database was the Alberta Health Care Insurance Plan Registry (it was also called the population registry) for the CHR for the fiscal years 1998/1999 to 2001/2002. This registry contained demographic information of health care recipients. Canada has a government-financed universal health insurance system. All permanent residents are covered by the provincial health insurance plan except for Registered First Nations, prison inmates, and members of the military and the Royal Canadian Mounted Police all of which are the responsibility of the federal government. All eligible Alberta residents are assigned a unique lifetime Personal Health Number (PHN). Therefore, PHN is an ideal variable for performing record linkage. The Alberta insurance registry is nearly complete and consistent, and is used as a proxy for the population of Alberta.

The second database was the Alberta Vital Statistics registry for fiscal years 1998/99 to 2001/2002. Information in the death registry was derived from the Death Registration form, medical certificate of death, and the medical examiner's certificate of death (where appropriate). The registry captured deaths that occurred within Alberta but misses Alberta residents who died out of Alberta. Provincial and territorial Vital Statistics registries on all deaths were submitted to Statistics Canada annually.

The third database was the hospital discharge abstracts for the fiscal years 1998/1999 to 2001/2002. Abstracts are filed for all inpatient discharges from all hospitals in the CHR. Professional coders reviewed inpatient charts and extracted data on PHN, demographics, diagnoses, procedures, physician specialty and status of alive or death.

### Common identifiers among three databases

We selected surname, first name, sex, and date of birth as our common identifiers because they were less likely to be changed over time, compared to other identifiers like address. We assessed three different combinations of four identifiers: (1) surname (i.e. surname at birth or marital surname as recorded in these three databases), sex and date of birth (i.e. month, day, and year of birth); (2) first name, sex, and date of birth; (3) surname, first name, sex and date of birth.

To perform record linkage, the common identifiers were formatted in the same way across the three databases, capitalizing letters, removing blanks and dashes. One common linkage problem with using the name variable as a common identifier was that an individual's name could be represented in many different ways, with alternate spellings, initials, abbreviations and shortened forms of names making the linkage difficult. To deal with this common linkage issue, a Soundex coding method was employed to identify linkage between names that fail to match due to variant spellings of the names in the two databases [13]. The Soundex algorithm associated numbers with different groups of consonants, producing a numeric code following the initial letter that was robust with respect to variations in names that sound alike.

Correct linkage among the linked records was assessed by checking whether the unique PHN from various sources was identical within the matched record. We excluded matched records without PHN in both files. Although this identifier is complete both in the hospital discharge data and population registry, only 70% of the records in the Vital Statistics registry had a valid PHN, because it was not a mandatory variable.

### Deterministic record linkage

#### Record linkage and correct linkage evaluation between vital statistics and population registry

**Figure 1:**
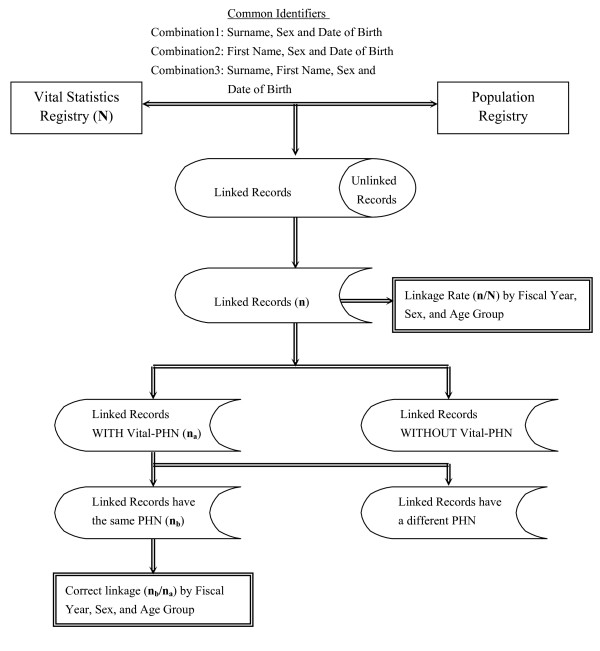
Matching Process between Vital Statistics registry and Population registry.

We linked the Vital Statistics registry with the population registry three times, using each of the three approaches (see Figure 1). In the process of linkage, the Vital Statistics registry was used as the master. The linkage rate between the population registry and Vital Statistics files was calculated using the number of records in the Vital Statistics registry file as the denominator (N in Figure 1) and the number of linked records as numerator (n in Figure 1). In the process of linkage, about 2% of Vital Statistics records were matched with more than one population registry record (i.e., duplicate records) for approach one, about 6% for approach two and less than 0.1% for approach three.

To assess the correct linkage, we checked whether the PHN obtained from the population registry was identical to the PHN recorded in the Vital Statistics registry among the linked records. Specifically we restricted the linked records to those with a valid PHN and checked the proportion of these records where the Vital-PHN matched the population-PHN. The Vital Statistics records with duplicate population registry records no matter whether there were PHNs or not, were defined as incorrect links. The correct linkage rate was calculated using the ratio of linked records with the matched PHNs over those with a valid Vital-PHN (i.e. the ratio of n_b _over n_a _in the Figure 1).

#### Record linkage and correct linkage evaluation between in-hospital death records and vital statistics files

**Figure 2:**
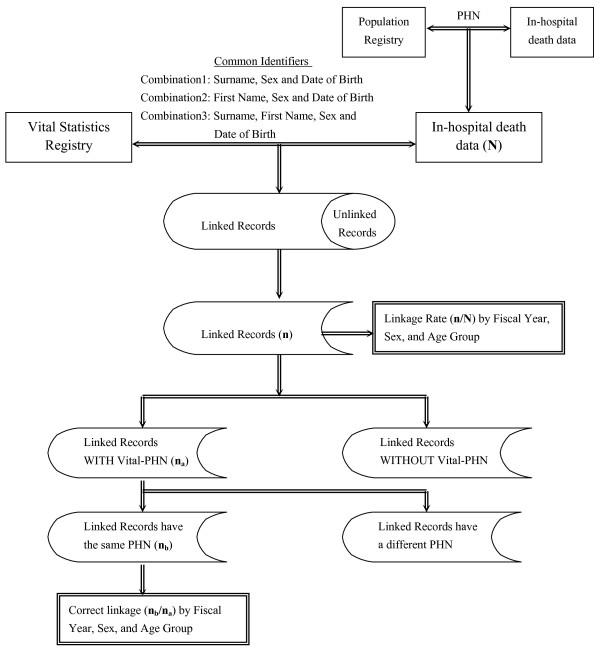
Matching Process between Vital Statistics registry and In-hospital death data.

We first selected inhospital death records in the hospital discharge data. Because our version of hospital discharge data did not contain names, we linked them with the population registry using PHNs that were present for all records in both files to retrieve the surname and first name from the population registry. In the matching process, the in-hospital death records were accepted as the master. We matched the in-hospital deaths to the Vital Statistics deaths using the three approaches, respectively (see Figure 2). The number of death records in the hospital discharge data (N in Figure 2) was used as a denominator to calculate the linkage rate. Among the three approaches, less than 0.1% of in-hospital death records had more than one match with Vital Statistics files. Likewise, those duplicate matches were defined as incorrect links. Correct linkage was assessed by checking whether the inhospital-PHN matched the Vital-PHN within the linked record. The correct linkage rate was obtained by the ratio of n_b _divided by n_a _(see Figure 2).

## Results

### Linkage and correct linkage rates between vital statistics and population registry

Table 1 presents the percentage of deaths in the Vital Statistics registry which can be linked with the population registry. Among the three approaches, approach one (surname, sex and date of birth) had the highest linkage rate (88.0% in 2001/02) compared to approach two (first name, sex and date of birth, 82.4% in 2001/02) and approach three (surname, first name, sex and date of birth, 79.5% in 2001/02).

For all three approaches, the linkage rate was similar between male and female in each fiscal year, but increased with age. For example, using approach one, the linkage rate was 39.7% for age 1 to 9 years and 90.0% for age 65 or older in fiscal year 2001/02. The linkage rate across fiscal years varied for age group of 1 to 39 years old and tended to increase for age groups of 40 years or older.

The correct linkage rate of record linkage among records that were linked between the Vital Statistics registry and population registry data for each approach is presented in Table 2. The correct linkage rate was 96.9% using approach one, 89.6% using approach two, and 98.5% using approach three in the 2001 fiscal year. The correct linkage rate showed an upward trend across fiscal years for all three approaches. The correct linkage rate was similar for both sex and age groups.

### Linkage and correct linkage rates between in-hospital death records and vital statistics files

Table 3 shows that the record linkage rate between in-hospital death records and Vital Statistics in 2001/02 was 93.1% using approach one, 85.0% using approach two, and 83.3% using approach three. For each record linkage approach, the linkage rate increased over fiscal years, was slightly higher for males than females, and did not vary much by age. In 2001/02, approach one had the lowest linkage rate for the 20 to 29 year age group (70.0%) but the highest rate for age 50 to 64 years (94.7%).

Table 4 shows the correct linkage rate among those linked between hospital discharge data and the Vital Statistics registry after excluding 29% to 43% of Vital Statistics records because of missing PHN values. The correct linkage rate was similar across three approaches, 98.9% using approach one in 2001/02, 98.8% using approach two and 99.1% using approach three. The correct linkage rate increased each fiscal year but was stable across sex and age.

## Discussion

This study assessed the record linkage and correct linkage rate using a deterministic record linkage method among three Canadian administrative databases. Our results showed that among the three combinations using the four possible identifiers, the combination of surname, sex and date of birth is the optimal choice in achieving the best deterministic linkage. Adding first name to the combination of identifiers increased the correct linkage rate no more than 1%, but at the cost of decreasing the linkage rate almost 10%. The linkage rate between the population and Vital Statistics registries when using the combination of surname, sex and date of birth was 88.0%; the correct linkage rate was 96.9%. For linkage between in-hospital death data and the Vital Statistics registry, the same combination achieved a linkage rate of 93.1% and a correct linkage rate of 98.9%. The linkage and correct linkage rates varied with the database being used and the age group, but not by sex.

The linkage and correct linkage rates depend on the databases being linked. The linkage between the population and Vital Statistics registries produced lower linkage and correct linkage rates than those between hospital discharge data and the Vital Statistics registry. One possible reason for this is that while completing death certificates for in-hospital deaths, hospital charts were consulted and personal information recorded in the chart was copied. Personal information in the chart is generally from the health insurance card, on which the Alberta health insurance plan prints PHN, full name, sex and date of birth. Thus more complete and accurate information might be included on death certificates for in-hospital deaths, thereby improving the linkage between Vital Statistics and hospital discharge data.

Vital Statistics records without PHN are likely to be persons who died out of hospital. Personal information for those deaths is from various sources, resulting in inconsistencies in personal information between Vital Statistics and the Alberta population registry. For persons who are not eligible for Alberta Health Insurance plan (such as inmates, travelers, visitors, expatriates, armed forces, and Royal Canadian Mounted Police), their PHNs would not be recorded in the Vital Statistics if they died in Alberta. However, such cases account for a small proportion of all deaths in Alberta.

The linkage rates between the Vital Statistics registry and hospital death do not vary much by age, but the linkage rate between the population registry and the Vital Statistics registry depends on age group. The linkage rate was significantly lower for the 1 to 9 year age group (ranging from 34.9% to 39.7% in 2001/02) than for 10 and over year age group (ranging from 47.0% to 90.0% in 2001/02). The difference in linkage rates may reflect less accurate information recorded for out-of-hospital deaths in younger persons. Records with the PHN may have more complete and accurate information on common identifiers than those without the PHN. In fact, we found the linkage rate between the population registry and Vital Statistics was higher for Vital Statistics records with PHN than for records without PHN (89.5% versus 81.0% in 2001/2002). There were more PHNs missing for children aged 1 to 9 years (42.9% in 2001/2002) than for individuals aged 10 or older (13.8% in 2001/02).

A successful deterministic linkage relies not only on the completeness of data, but also on choosing an appropriate combination of common identifiers. In our study, a combination of surname, sex and date of birth has the highest linkage rate and second highest correct linkage rate. The combination of first name, sex and date of birth generated relatively low linkage and correct linkage rates. The combination of all four identifiers (first, surname, sex and date of birth) resulted in an even lower linkage rate with little increase in the correct linkage rate. One possibility is that alternate spellings, initials, abbreviations and shortened forms of first names are common in the database. Therefore we recommend the use surname, sex and date of birth as common identifiers in linking databases deterministically when a unique identifier is not available.

To further improve correct record linkage, other potential indicators, such as residence address or postal code, should be considered. In our study, only 67% to 77% of records in the Vital Statistics registry during 1998 and 2001 contained information on the unique identifier of personal health number, while 84% to 96% had information on postal codes. The linkage rates might be increased taking the strategy of first matching records using unique identifiers, and then matching remaining records using a combination of surname, sex and date of birth.

This study has four major limitations. First, we assessed record linkage using administrative databases in a Canadian health region. The results might not be conceptually generalizable to other Canadian regions or other countries because the quality of administrative data may vary across geographical areas and institutions. Secondly, records without PHN may have less complete and accurate information on common identifiers than those with PHN. Therefore the higher the rate of missing PHNs is, the more likely the linkage rate is to be lower. We selected linked records with PHNs only to assess correct linkage rate. The potential selection bias may cause the correct linkage rate to be overestimated, particularly for children aged 1 to 9 since they have more missing PHNs than those aged 10 or older in Vital Statistics data. Thirdly, we excluded deaths with unknown residence area information and missed residents of the region who died out of Alberta, possibly leading to overestimates of our linkage rate if personal information on these deaths was less complete than those we analyzed. Fourthly, our linkage rate may be applicable to linking nested databases; one database contains all records of other database. In our study, all Vital Statistics Registry records are expected to appear in the Population Registry, and all in-hospital death records are expected to be present in the Vital Statistics Registry. Correct linkage could be assessed by four measures: true-link, false-link, true-nonlink and false-nonlink. Assessment of these four types requires a unique identifier present in both databases to establish the "gold standard". Our study addressed one question: what is correct linkage rate among links through deterministic record linkage?

## Conclusion

Our study findings suggest that deterministic record linkage using three basic indicators (i.e., surname, sex and date of birth) appears to generate the highest linkage rate among three commonly used databases in health service research, namely the population registry, hospital discharge data and the Vital Statistics registry. The matched records appear to be highly accurate. However, the linkage and correct linkage rates appear to be influenced by type of database and age, but not by sex.

## Competing interests

The author(s) declare that they have no competing interests.

## Authors' contributions

BL contributed to the study design, statistical analysis, interpretation and writing of the manuscript. HQ contributed to the study design, the interpretation, and writing of the manuscript. AF helped data-analysis and interpretation and editing and proving the manuscript. ML contributed to the data interpretation and writing of the manuscript.

## Pre-publication history

The pre-publication history for this paper can be accessed here:



## Figures and Tables

**Table 1:** Linkage Rate (%) between Vital Statistics registry and Population registry

A combination of common identifiers		1998/99 (N = 4717)	1999/00 (N = 5024)	2000/01 (N = 5299)	2001/02 (N = 5182)
Surname, Sex and Date of Birth					
Total		85.6	85.6	87.2	88.0
Sex:	Female	85.0	86.0	86.9	88.2
	Male	86.2	85.3	87.5	87.7
Age:	1 to 9 years	47.2	26.0	32.7	39.7
	10 to 19 years	70.5	77.8	67.4	66.7
	20 to 29 years	75.0	69.7	64.0	66.7
	30 to 39 years	86.4	80.1	80.9	82.8
	40 to 49 years	84.1	87.6	87.4	85.9
	50 to 64 years	83.2	85.6	87.2	88.0
	Over 65 years	87.2	87.5	89.0	90.0
First Name, Sex and Date of Birth					
Total		78.8	79.5	81.2	82.4
Sex:	Female	76.6	78.0	80.1	81.7
	Male	80.9	81.0	82.2	83.2
Age:	1 to 9 years	41.5	26.0	34.6	36.5
	10 to 19 years	59.1	68.9	61.2	50.0
	20 to 29 years	70.0	71.6	67.4	73.1
	30 to 39 years	87.6	80.1	77.8	79.1
	40 to 49 years	83.7	83.0	87.0	84.0
	50 to 64 years	76.8	81.2	82.4	83.4
	Over 65 years	79.5	80.3	81.8	83.7
Surname, First Name, Sex and Date of Birth					
Total		75.5	76.0	78.7	79.5
Sex:	Female	73.0	74.5	77.1	78.9
	Male	77.8	77.5	80.1	80.0
Age:	1 to 9 years	41.5	22.1	32.7	34.9
	10 to 19 years	52.3	60.0	51.0	47.0
	20 to 29 years	63.0	60.6	59.3	62.8
	30 to 39 years	79.9	73.3	71.6	71.6
	40 to 49 years	73.9	76.3	81.1	78.1
	50 to 64 years	72.2	76.9	78.8	79.4
	Over 65 years	77.2	77.8	80.2	81.5

**Table 2:** Correct Linkage Rates (%) among Records Matched between Vital Statistics registry and Population registry

A combination of common identifiers		1998/99	1999/00	2000/01	2001/02
Surname, Sex and Date of Birth		(N = 2620)	(N = 3014)	(N = 3445)	(N = 3530)
Total		96.1	96.3	96.9	96.9
Sex:	Female	96.4	96.2	97.2	97.3
Age:	Male	95.8	96.4	96.5	96.6
	1 to 9 years	100.0	92.3	100.0	93.3
	10 to 19 years	86.7	94.7	100.0	96.0
	20 to 29 years	96.2	87.9	87.5	90.5
	30 to 39 years	90.9	92.4	90.8	94.8
	40 to 49 years	93.9	93.5	96.0	94.5
	50 to 64 years	93.8	95.9	94.5	96.0
	Over 65 years	96.8	96.8	97.5	97.4
		
First Name, Sex and Date of Birth		(N = 2405)	(N = 2777)	(N = 3202)	(N = 3298)
Total		89.3	89.1	89.3	89.6
Sex:	Female	89.5	90.0	89.3	90.5
	Male	89.1	88.1	89.3	88.7
Age:	1 to 9 years	100.0	91.7	70.0	85.7
	10 to 19 years	76.9	87.5	73.7	84.2
	20 to 29 years	91.7	80.0	86.4	76.0
	30 to 39 years	74.2	74.6	67.7	73.7
	40 to 49 years	71.7	66.4	68.2	71.6
	50 to 64 years	82.9	88.5	82.0	85.2
	Over 65 years	91.9	91.9	92.8	92.2
		
Surname, First Name, Sex and Date of Birth		(N = 2311)	(N = 2672)	(N = 3120)	(N = 3188)
Total		97.3	97.6	98.2	98.5
Sex:	Female	97.6	97.8	98.1	98.3
	Male	97.1	97.4	98.2	98.7
Age:	1 to 9 years	100.0	100.0	100.0	100.0
	10 to 19 years	91.7	100.0	100.0	94.1
	20 to 29 years	100.0	96.2	100.0	100.0
	30 to 39 years	96.6	98.3	96.7	98.2
	40 to 49 years	95.8	96.3	99.3	98.2
	50 to 64 years	95.3	97.7	98.0	98.5
	Over 65 years	97.8	97.6	98.1	98.5

**Table 3:** Linkage Rates (%) between Vital Statistics registry and In-hospital death data

A combination of common identifiers		1998/99 (N = 1639)	1999/00 (N = 1637)	2000/01 (N = 1707)	2001/02 (N = 1779)
Surname, Sex and Date of Birth					
Total		89.6	89.3	91.5	93.1
Sex:	Female	88.0	88.3	90.8	93.8
	Male	90.9	90.1	92.0	92.4
Age:	1 to 9 years	100.0	100.0	88.9	77.8
	10 to 19 years	100.0	85.7	88.9	92.3
	20 to 29 years	88.2	80.0	78.6	70.0
	30 to 39 years	92.1	80.6	83.9	85.2
	40 to 49 years	87.7	90.1	90.7	88.2
	50 to 64 years	85.5	87.1	91.1	94.7
	Over 65 years	90.2	90.0	91.9	93.5
First Name, Sex and Date of Birth					
Total		80.1	82.3	82.8	85.0
Sex:	Female	76.6	80.8	82.7	83.3
	Male	83.2	83.6	82.9	86.6
Age:	1 to 9 years	77.8	83.3	100.0	88.9
	10 to 19 years	70.0	78.6	66.7	76.9
	20 to 29 years	94.1	70.0	78.6	80.0
	30 to 39 years	89.5	86.1	74.2	74.1
	40 to 49 years	80.3	79.0	84.0	83.9
	50 to 64 years	77.9	80.6	82.1	85.1
	Over 65 years	80.2	82.9	83.1	85.4
Surname, First Name, Sex and Date of Birth					
Total		78.2	79.8	81.7	83.3
Sex:	Female	74.2	78.1	81.2	81.9
	Male	81.6	81.4	82.2	84.5
Age:	1 to 9 years	77.8	83.3	88.9	77.8
	10 to 19 years	70.0	71.4	55.6	76.9
	20 to 29 years	88.2	60.0	78.6	60.0
	30 to 39 years	86.8	77.8	71.0	74.1
	40 to 49 years	76.5	77.8	80.0	81.7
	50 to 64 years	73.6	77.6	81.3	83.7
	Over 65 years	78.8	80.8	82.3	83.7

**Table 4:** Correct linkage Rates (%) among Matched Records between Vital Statistics registry and In-hospital death data

A combination of common identifiers		1998/99	1999/00	2000/01	2001/02
Surname, Sex and Date of Birth		(N = 1056)	(N = 1102)	(N = 1253)	(N = 1411)
Total		98.1	97.1	98.7	98.9
Sex:	Female	98.4	96.7	98.8	98.7
	Male	97.9	97.4	98.6	99.2
Age:	1 to 9 years	100.0	100.0	100.0	100.0
	10 to 19 years	100.0	88.9	100.0	85.7
	20 to 29 years	100.0	90.9	100.0	100.0
	30 to 39 years	100.0	95.0	94.4	100.0
	40 to 49 years	96.3	98.3	98.1	100.0
	50 to 64 years	95.9	97.3	99.5	98.7
	Over 65 years	98.5	97.2	98.7	99.0
		
First Name, Sex and Date of Birth		(N = 960)	(N = 1019)	(N = 1142)	(N = 1286)
Total		97.8	96.8	98.4	98.8
Sex:	Female	98.1	96.3	98.7	98.7
	Male	97.6	97.2	98.2	98.9
Age:	1 to 9 years	100.0	100.0	100.0	100.0
	10 to 19 years	100.0	88.9	75.0	80.0
	20 to 29 years	100.0	88.9	100.0	100.0
	30 to 39 years	100.0	95.0	94.4	100.0
	40 to 49 years	95.9	97.9	97.9	100.0
	50 to 64 years	95.6	97.1	98.8	98.6
	Over 65 years	98.2	96.8	98.5	98.8
		
Surname, First Name, Sex and Date of Birth		(N = 936)	(N = 990)	(N = 1126)	(N = 1261)
Total		97.9	96.8	98.7	99.1
Sex:	Female	98.1	96.3	98.9	98.8
	Male	97.7	97.2	98.5	99.3
Age:	1 to 9 years	100.0	100.0	100.0	100.0
	10 to 19 years	100.0	87.5	100.0	80.0
	20 to 29 years	100.0	87.5	100.0	100.0
	30 to 39 years	100.0	94.7	94.1	100.0
	40 to 49 years	95.8	97.9	97.8	100.0
	50 to 64 years	95.4	97.0	99.4	98.5
	Over 65 years	98.3	96.9	98.6	99.2
